# MARITAL HISTORY AND OLDER ADULTS’ RELATIONSHIPS WITH ADULT CHILDREN

**DOI:** 10.1093/geroni/igae098.1535

**Published:** 2024-12-31

**Authors:** Won Choi

**Affiliations:** University of Texas at Austin, Chicago, Illinois, United States

## Abstract

The diverse marital experiences of older adults today may shape their relationship with adult children. This study examined whether marital status (i.e., continuously married, remarried, divorced, widowed) and marital transitions (i.e., age at first marriage, number of marital dissolutions, time spent unmarried after first marriage) are associated with the availability, quality, and stability of older adults’ social network ties to their children. This study used retrospective marital histories and longitudinal egocentric network data from the National Social Life, Health, and Aging Project conducted between 2005 and 2011 (N = 1,873). Multivariate regression models were used to estimate the relationship between marital factors and older adults’: (1) likelihood of listing their children as social network members in 2005/2006, (2) frequency of contact with and (3) emotional closeness to child network members, and (4) likelihood of stop listing their children as network members by 2010/2011. Results showed that remarried older adults were less likely to name a child network member, reported less frequent contact with child network members, and were more likely to stop naming their children as network members over time compared to continuously married older adults. Conversely, previously married individuals (i.e., divorced or widowed) were more likely to name a child network member and reported greater emotional closeness to child network members than continuously married older adults. Marital transitions partly explained these differences. The findings highlight the importance of considering past and current marital experiences in understanding parent-child relationships in later life.

